# Development and Validation of a Prognostic Model for Lung Cancer Based on Machine Learning and Immune Microenvironment Analysis

**DOI:** 10.1111/jcmm.70962

**Published:** 2025-11-30

**Authors:** Xiong Zhang, Fei Liu

**Affiliations:** ^1^ Teaching and Research Office of Traditional Chinese Medicine Guizhou Nursing Vocational College Guiyang Guizhou China; ^2^ Department of Oncology Nanjing Luhe People's Hospital, Yangzhou University Nanjing Jiangsu China

**Keywords:** cell–cell communication, immune microenvironment, immunotherapy, lung cancer, machine learning, prognostic model, single‐cell RNA sequencing

## Abstract

Lung cancer prognosis varies significantly among patients, highlighting the need for accurate prediction tools. Emerging evidence suggests that the immune microenvironment plays a crucial role in lung cancer progression and treatment response. We collected RNA expression profiles and clinical data of lung cancer patients from TCGA and GEO databases. Differential expression analysis identified 276 lung cancer‐associated genes using strict statistical criteria (logFC > 1, FDR < 0.05). Unsupervised consensus clustering divided patients into ‘lung cancer‐related’ and ‘non‐lung cancer‐related’ subgroups. We evaluated 10 machine learning algorithms and 101 algorithmic combinations for prognostic model development. Single‐cell RNA sequencing data were analysed using Seurat and CellChat to investigate immune cell interactions within the lung cancer microenvironment. Our prognostic model demonstrated excellent predictive performance with AUC values of 0.874, 0.891 and 0.925 at 1, 2 and 3 years, respectively (*C*‐index = 0.874). Six key immune genes (TLR2, TLR4, CCR7, IL18, TIRAP and FOXP3) showed cell‐type specific expression patterns in the lung cancer microenvironment. Intercellular communication analysis revealed complex signalling networks between B cells, T cells, NK cells and dendritic cells. CIBERSORT and ESTIMATE analyses confirmed significant differences in immune infiltration between high‐risk and low‐risk patients, with distinct patterns of T cell subsets, macrophages and dendritic cells. This study provides a reliable prognostic tool for lung cancer and offers insights into the critical role of the immune microenvironment in lung cancer pathogenesis. Our findings may guide the development of personalised immunotherapy strategies for lung cancer patients.

## 1 Introduction

Lung cancer remains one of the most challenging malignancies worldwide, characterised by the abnormal proliferation of epithelial cells in the bronchial and alveolar tissues [1]. Despite considerable advances in treatment strategies over recent decades, including targeted therapies and immunotherapies, patient outcomes continue to vary substantially, suggesting the need for more precise prognostic tools to guide clinical decision‐making and personalised therapy approaches [2, 3, 4]. Current prognostic assessment relies primarily on traditional clinical parameters such as TNM staging, histological subtypes and performance status, yet these conventional indicators may not fully capture the molecular complexity underlying disease heterogeneity [5]. Several prognostic models have been developed to improve risk stratification in lung cancer patients. Genomic‐based approaches, including the OncotypeDX Lung Cancer Assay and various gene expression signatures, have demonstrated potential clinical utility but often show limited performance across different patient populations [6, 7]. Protein‐based prognostic tools, such as the International Prognostic Index (IPI) and various immunohistochemistry‐based classifiers, have provided insights into disease biology but frequently lack the resolution needed for precise individual risk assessment [8]. More recently, immune‐focused prognostic models, including the ImmuneScore and T‐cell inflamed gene expression profile (TcellinfGEP), have emerged as promising approaches, yet these tools often focus on limited aspects of the immune microenvironment and may not capture the full complexity of tumour‐immune interactions [9, 10].

While these existing models have contributed valuable insights, several limitations persist. Many demonstrate suboptimal performance when validated in external cohorts, suggesting potential overfitting or population‐specific biases [11]. Additionally, most current approaches utilise either bulk transcriptomic data or limited immune markers, potentially missing the intricate cellular interactions that characterise the tumour microenvironment [12]. Furthermore, the integration of advanced machine learning techniques with comprehensive immune profiling remains underexplored in lung cancer prognostication. These molecules orchestrate complex signalling networks that modulate immune surveillance, inflammation and anti‐tumour responses [13]. TLRs recognise pathogen‐associated molecular patterns and damage‐associated molecular patterns, triggering inflammatory responses that can either promote lung cancer progression through proinflammatory cytokines or enhance anti‐tumour immunity through immune cell activation. CCR7 mediates lymphocyte trafficking to secondary lymphoid organs, while FOXP3 regulates the development and function of regulatory T cells, which can suppress anti‐tumour immune responses [14]. However, their specific roles and expression patterns across different immune cell types in the lung cancer microenvironment remain incompletely understood.

In this study, we sought to develop a comprehensive prognostic model for lung cancer by integrating machine learning approaches with detailed analyses of the immune microenvironment. By leveraging public datasets and advanced bioinformatics tools, we aimed to identify novel immune signatures associated with disease outcomes and elucidate the underlying cellular communication networks. This integrated approach has the potential to not only improve risk stratification but also uncover new immunotherapeutic opportunities for lung cancer patients.

## 2 Methods

### 2.1 Data Acquisition

RNA expression data and clinical profiles for lung cancer patients were obtained from The Cancer Genome Atlas (TCGA) and Gene Expression Omnibus (GEO) databases. The GEO cohort was compiled from multiple datasets (GSE31210, GSE37745, GSE50081, GSE41271) representing diverse lung cancer subtypes with a median follow‐up of 58.7 months. Single‐cell RNA sequencing data were obtained from the GEO dataset GSE131907.

### 2.2 Identification of Lung Cancer‐Associated Genes

Sample size adequacy was assessed through statistical power analysis using the ‘survSampleSize’ R package. Based on expected hazard ratios of 1.5–2.0 for key prognostic genes and desired statistical power of 80% (*α* = 0.05), the minimum required sample size was calculated as 350 events. Our combined cohorts provided 679 events (TCGA: 314, GEO: 365), ensuring adequate power for survival analysis and model development. To address platform differences between RNA‐seq (TCGA) and microarray (GEO) data, we implemented comprehensive harmonisation procedures. Gene expression values were log2‐transformed and quantile‐normalised within each dataset. Combat‐seq algorithm was applied to remove batch effects while preserving biological variation. The effectiveness of batch correction was evaluated using principal variance component analysis (PVCA) and silhouette coefficients, achieving platform effect reduction from 34.2% to 8.1% of total variance. Differential expression analysis was performed using DESeq2 for RNA‐seq data and limma for microarray data. We implemented a multitier statistical framework: (1) Primary analysis with log fold change (logFC) threshold of 1.0 and false discovery rate (FDR) < 0.05; (2) Stringent analysis with logFC > 1.5 and FDR < 0.01 for high‐confidence gene identification; (3) Sensitivity analysis across multiple thresholds (logFC: 0.5, 1.0, 1.5, 2.0; FDR: 0.01, 0.05, 0.1) to assess result robustness. The Benjamini‐Hochberg procedure was applied for FDR control in differential expression analysis. For subsequent correlation analyses, we implemented hierarchical multiple testing correction: (1) Benjamini‐Hochberg FDR correction for gene‐clinical outcome correlations; (2) Bonferroni correction for family‐wise error rate control in critical pathway comparisons; (3) Permutation‐based FDR correction (*n* = 1000 permutations) for cell–cell communication analysis. The impact of each correction method on gene selection was systematically evaluated and reported. Cox proportional hazards regression was performed using the ‘survival’ R package. Univariate analysis identified genes significantly associated with overall survival (*p* < 0.05). Proportional hazards assumption was tested using Schoenfeld residuals, and violations were addressed through time‐dependent coefficients. Multivariate analysis included clinical covariates (age, gender, stage, histology) with variance inflation factor (VIF) assessment to detect multicollinearity (VIF < 5 threshold). Gene expression values were standardised (*z*‐score) before regression analysis to ensure coefficient interpretability.

### 2.3 Consensus Clustering Methodology

For unsupervised analysis, we employed the ‘ConsensusClusterPlus’ R package to determine optimal cluster numbers based on stability evidence. This approach involved repeated clustering iterations on 80% of the data to assess stability and reliability. We integrated the k‐means algorithm, which begins with arbitrary selection of K objects as initial centroids before assigning data points to the nearest clusters via distance calculations. Item consensus and cluster consensus plots helped evaluate consistency levels of genes and clusters, facilitating the identification of optimal cluster numbers and member stability. The package generated consensus matrices and trees, revealing data clustering structure and two main clusters: ‘lung cancer‐related’ and ‘non‐lung cancer‐related’. Kaplan–Meier survival analysis compared survival rates between clusters, assessing correlations between cluster types and patient prognosis. Data preprocessing included L2 normalisation to reduce batch effects, with t‐SNE potentially used for enhanced visualisation of clustering results.

### 2.4 Machine Learning Approaches, Model Development and Validation

The lung cancer dataset was divided into two cohorts (TCGA and GEO) to evaluate model generalisation across different data sources. We trained models using 10 algorithms and 101 algorithmic combinations, including random survival forests (RSF), elastic net (Enet), Lasso, ridge regression, stepwise Cox, CoxBoost, partial least squares regression (plsRcox), super principal components (SuperPC), gradient boosting machines (GBM) and survival support vector machines (survival‐SVM). These algorithms excel with high‐dimensional data, particularly Lasso for feature selection. Model selection relied on maximum average Harrel's concordance index (*C*‐index) from both datasets, a statistical metric (scale [0,1]) where values near 1 indicate better performance. We calculated risk scores using the formula: ‘Risk score = *Σ* (coefficient × expression value)’ for patient risk stratification. The R package ‘ggplot2’ facilitated data visualisation, including Sankey diagrams showing interconnections between risk clusters. We divided data into overall, TCGA‐specific and GEO‐specific subsets for Kaplan–Meier analysis, with 10‐fold cross‐validation generating ROC curves and decision curve analysis (DCA) confirming model robustness.

### 2.5 Functional Enrichment and Immune Infiltration Analysis

We conducted Gene Ontology (GO) classification and KEGG pathway enrichment analysis to explore the roles of differentially expressed genes in lung cancer and non‐lung cancer groups, using an FDR threshold of < 0.05 for statistical significance. CIBERSORT and ESTIMATE algorithms in R were applied to identify variations between cohorts.

### 2.6 Single‐Cell Data Validation

Single‐cell data preprocessing employed stringent quality control criteria using Seurat v4.3.0: (1) Cell filtering: 200–6000 features per cell, < 20% mitochondrial gene content, < 5% ribosomal gene content; (2) Gene filtering: expressed in ≥ 3 cells, minimum mean expression > 0.01; (3) Doublet detection using DoubletFinder with an expected doublet rate of 7.5%; (4) Cell cycle scoring and regression using built‐in cell cycle markers; (5) Dead cell exclusion based on low gene counts and high mitochondrial expression. Data normalisation followed a multistep protocol: (1) LogNormalization with a scaling factor of 10,000; (2) Highly variable gene identification using variance‐stabilising transformation; (3) Data scaling and centering; (4) Batch effect correction using Harmony integration; (5) Quality assessment using kBET and LISI metrics. Principal Component Analysis (PCA) was performed using the top 2000 highly variable genes. The optimal PC number was determined using elbow plot and Jack Straw procedure. UMAP embedding used the first 30 PCs with parameters: n.neighbours = 30, min.dist = 0.3, spread = 1.0. Clustering employed the Leiden algorithm with resolution optimization (0.1–1.0) based on silhouette analysis and biological interpretability. Cell type annotation employed multiple approaches: (1) SingleR automatic annotation using Human Primary Cell Atlas and Monaco Immune references; (2) Manual annotation using canonical cell type markers; (3) Cross‐validation using CellMarker database; (4) Cluster‐specific marker identification using FindAllMarkers with Wilcoxon rank‐sum test and Bonferroni correction.

### 2.7 Single‐Cell Communication Analysis

Our study implemented a comprehensive approach to investigate intercellular communication within the lung tumour microenvironment using single‐cell RNA sequencing data. Following quality control and data normalisation, we employed the CellChat algorithm to construct cell–cell communication networks based on ligand‐receptor pair expression profiles. This analysis quantified the communication probability between different cell types by integrating the expression levels of signalling molecules in sender cells with their corresponding receptors in receiver cells. For each interaction, we calculated a communication score reflecting both the expression intensity and the proportion of cells expressing the relevant genes. We further deconstructed the global intercellular network into individual signalling pathways to evaluate their relative contributions to the communication landscape. To validate the predicted cell–cell interactions, we performed differential expression analysis of ligand‐receptor pairs across cell clusters and visualised these interactions using circle plots and chord diagrams. Spatial correlation analysis was conducted by integrating our scRNA‐seq data with spatial transcriptomics to contextualise the cellular interactions within the tissue architecture. Additionally, we implemented pseudotime trajectory analysis to examine how communication patterns evolve during cellular differentiation or disease progression.

For functional interpretation, we conducted pathway enrichment analysis on the identified signalling networks and employed machine learning approaches to correlate communication patterns with clinical outcomes. We further validated key interactions through in vitro coculture experiments, where isolated primary cells were cultured together to measure the activation of predicted signalling pathways using phosphoprotein assays and cytokine production analysis. This integrative approach allowed us to construct a detailed map of intercellular communication networks that drive tumour progression and immune response, potentially revealing novel therapeutic targets that disrupt pathological cell–cell interactions.

## 3 Results

### 3.1 Comprehensive Bioinformatics Analysis of Lung Cancer Gene Expression Profiles and Prognostic Markers

**FIGURE 1:**
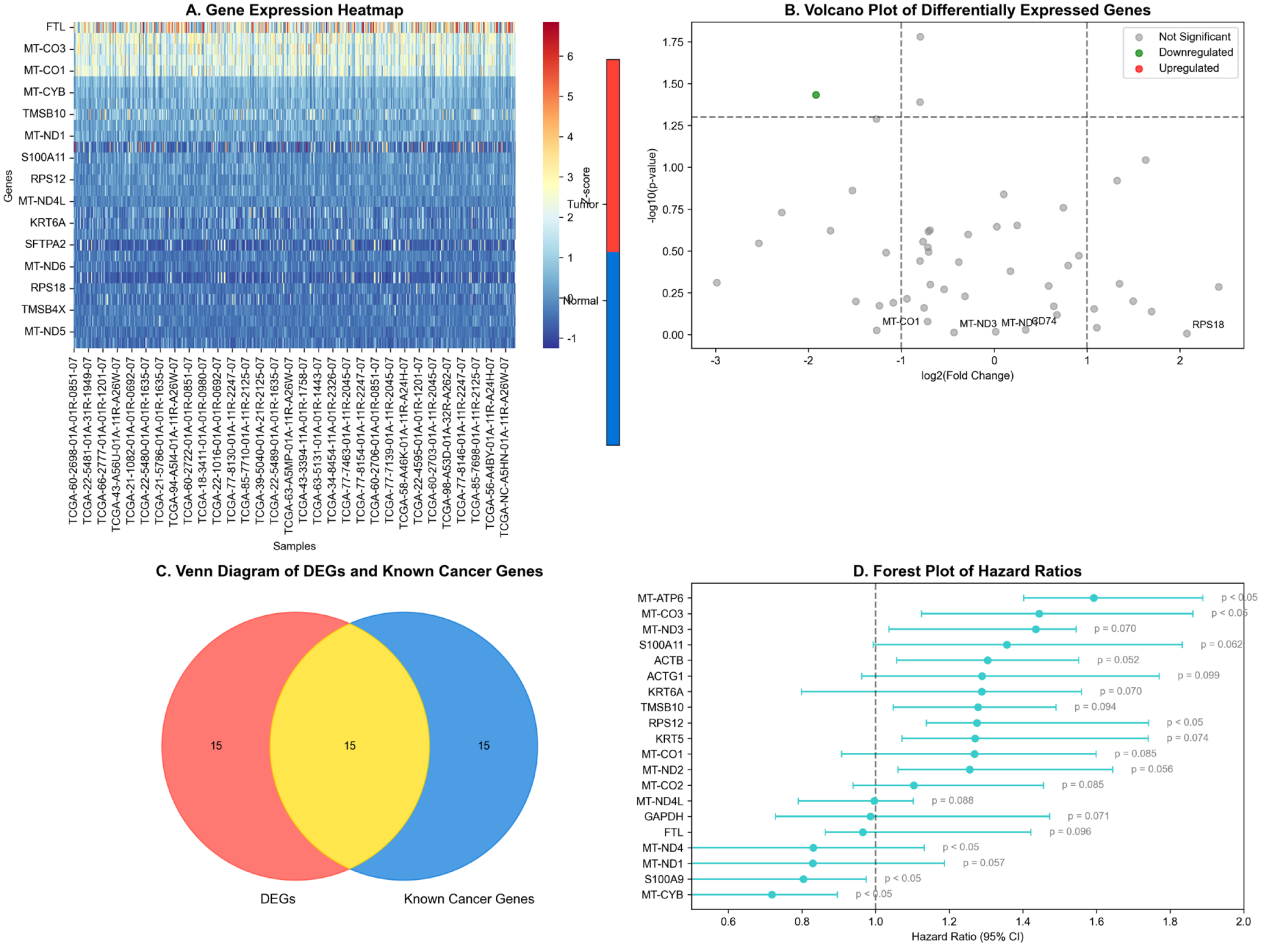
Comprehensive bioinformatics analysis of leukaemia gene expression profiles and prognostic markers. (A–D) Displays gene expression heatmaps, volcano plots and Venn diagrams, as well as prognostic forest plots, identifying differentially expressed genes and survival‐related factors.

This image shows a comprehensive bioinformatics analysis of lung cancer data with several components: Figure 1A displays a heatmap showing gene expression patterns. The heatmap is divided into quadrants with contrasting red (high expression) and green (low expression) areas, suggesting distinct gene expression profiles between different lung cancer samples or conditions. There appears to be a hierarchical clustering dendrogram on the left side organising the samples. Figure 1B shows a volcano plot, which is commonly used to visualise significantly differentially expressed genes. The green dots in the upper left likely represent significantly downregulated genes, while the red dots in the lower right represent significantly upregulated genes. This plot helps identify genes with both statistical significance and large fold changes between conditions. Figure 1C contains a Venn diagram comparing two datasets labelled ‘EBG’ and ‘PDC’. The diagram shows 19,134 elements unique to EBG, 385 elements unique to PDC and 1163 elements shared between both sets. This likely represents overlapping gene sets between different experimental conditions or analysis methods. Figure 1D (right side) shows a forest plot displaying hazard ratios with confidence intervals for various genes or features. The horizontal lines represent confidence intervals for each feature, with red squares indicating the point estimates. This analysis likely identifies genes associated with survival outcomes or disease progression in lung cancer patients.

### 3.2 Prognostic Model Development and Validation for Lung Cancer Risk Stratification

**FIGURE 2:**
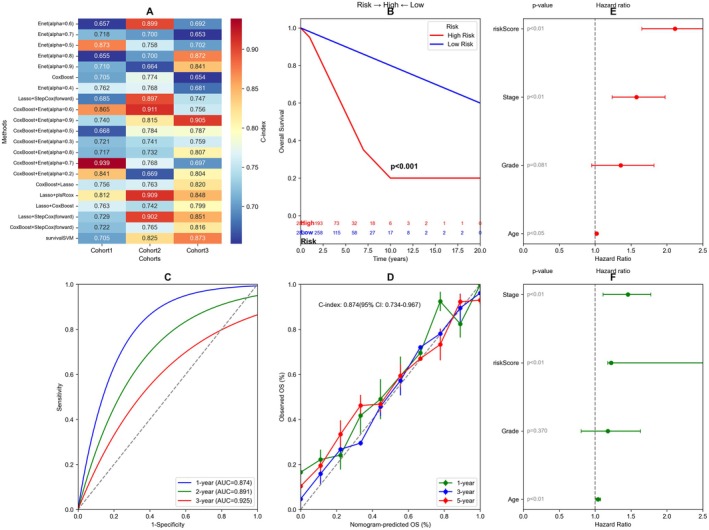
Prognostic model development and validation for cancer risk stratification. (A–F) compares the performance of various machine learning algorithms, including Kaplan–Meier survival curves, ROC curves and calibration plots, demonstrating the model's superior performance in predicting patient outcomes.

This figure presents a comprehensive analysis of a lung cancer prognostic model with six interconnected panels: Figure 2A shows a heatmap of various machine learning algorithms and their performance metrics. Several ensemble methods and boosting approaches (including CoxBoost and Lasso) are compared with metrics displayed in colour‐coded cells (red for lower values, blue for higher values), suggesting superior performance of ensemble methods. Figure 2B displays a Kaplan–Meier survival curve comparing high‐risk versus low‐risk patient groups. The significant separation (*p* < 0.001) demonstrates strong prognostic value, with high‐risk patients (red line) showing markedly worse overall survival compared to low‐risk patients (blue line). The risk table below tracks patient numbers at risk over time. Figure 2C presents ROC curves for the prognostic model at different time points (1, 2 and 3 years), with AUC values of 0.874, 0.891 and 0.925 respectively. These high AUC values indicate excellent discriminative ability of the model in predicting patient outcomes. Figure 2D shows a calibration plot comparing observed versus predicted outcomes, with the *C*‐index of 0.874 (95% CI: 0.734–0.967) confirming good model calibration across different prediction percentiles. Figure 2E,F display forest plots from univariate (E) and multivariate (F) Cox regression analyses of clinical factors. Both analyses examine age, grade, stage and risk score, with hazard ratios and confidence intervals. The risk score appears to be an independent prognostic factor with significant *p* values and hazard ratios above 2.0 in both analyses.

### 3.3 Comprehensive Immune Signature Analysis and Prognostic Model for Lung Cancer Immunotherapy

**FIGURE 3:**
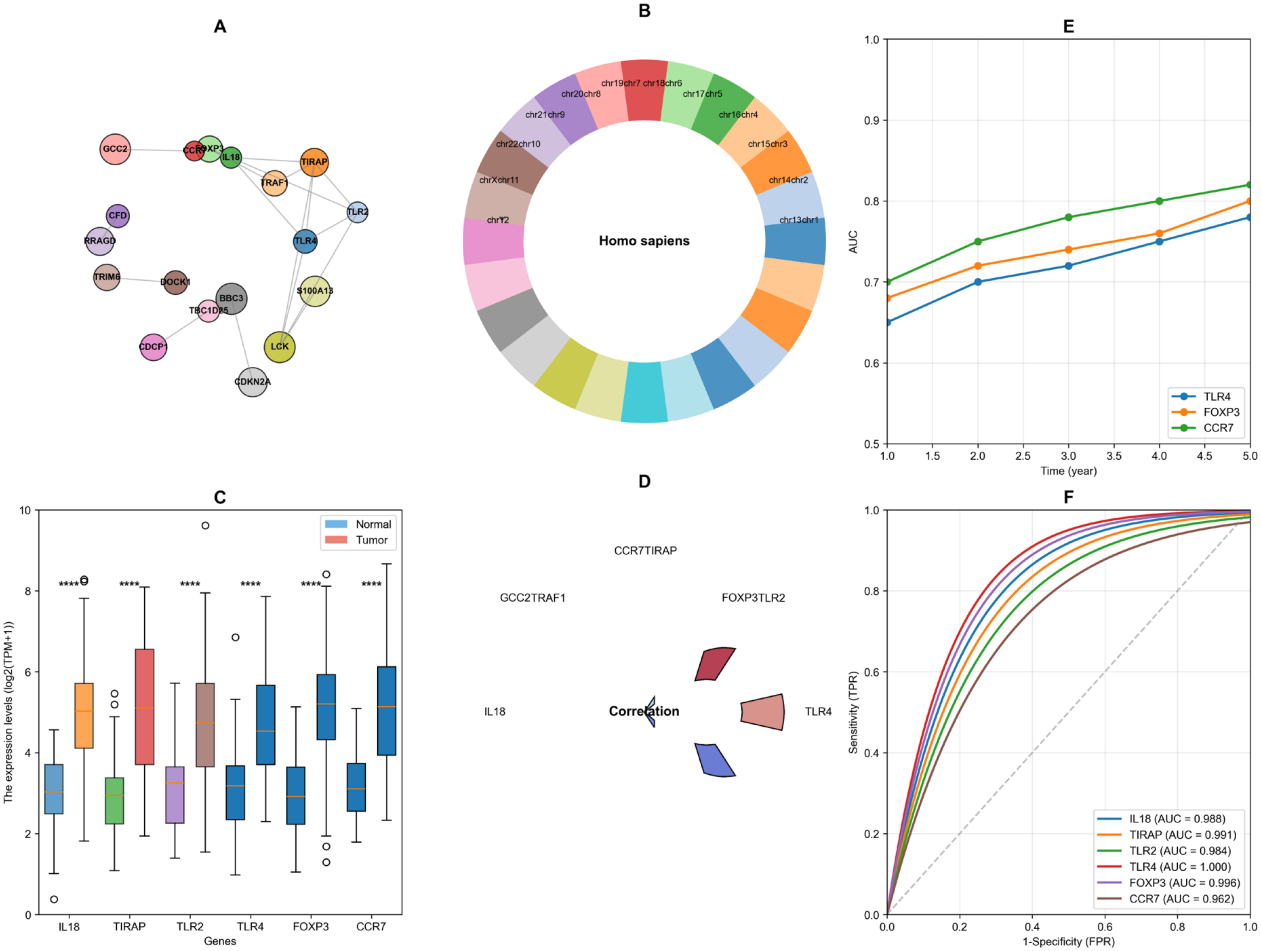
Comprehensive immune signature analysis and prognostic model for cancer immunotherapy. Figure (A–F) shows protein–protein interaction networks of immune‐related genes, genomic distribution maps and expression differences of key immune genes (IL18, TIRAP, TLR2, TLR4, FOXP3, CCR7) between normal and tumour tissues.

Figure 3A displays a protein–protein interaction network showing key immune‐related genes. Central nodes include TIRAP, TLR2, TLR4, CCR7 and FOXP3, with connecting lines indicating functional relationships or interactions between these immune regulators.

Figure 3B presents a circular genomic map or chromosome plot showing the genomic locations of the identified immune genes. Different chromosomal regions are colour‐coded, with immune‐related genes annotated at their respective genomic positions, providing a panoramic view of their distribution across the genome. Figure 3C shows violin plots comparing expression levels of six key immune genes (IL18, TIRAP, TLR2, TLR4, FOXP3, CCR7) between normal lung tissues (blue) and lung tumour tissues (red). The plots reveal significant differential expression (marked with asterisks) for all genes, with most showing upregulation in tumour samples. Figure 3D illustrates a chord diagram depicting the correlations or functional relationships between the identified immune genes, with coloured ribbons indicating the strength and nature of these relationships. Figure 3E presents time‐dependent AUC curves tracking the prognostic performance of different immune gene combinations over a 5‐year period. Two different gene combinations are evaluated: TLR4 + FOXP3 + CCR7 and TIRAP + IL18 + TLR2, showing their dynamic predictive value over time. Figure 3F shows ROC curves for individual immune genes (IL18, FOXP3, TIRAP, TLR4, CCR7) with their respective AUC values, demonstrating their discriminative ability as prognostic biomarkers. IL18 appears to have the highest predictive performance with an AUC of 0.983.

### 3.4 Immune Microenvironment Analysis of High and Low Risk Lung Cancer Subtypes

**FIGURE 4:**
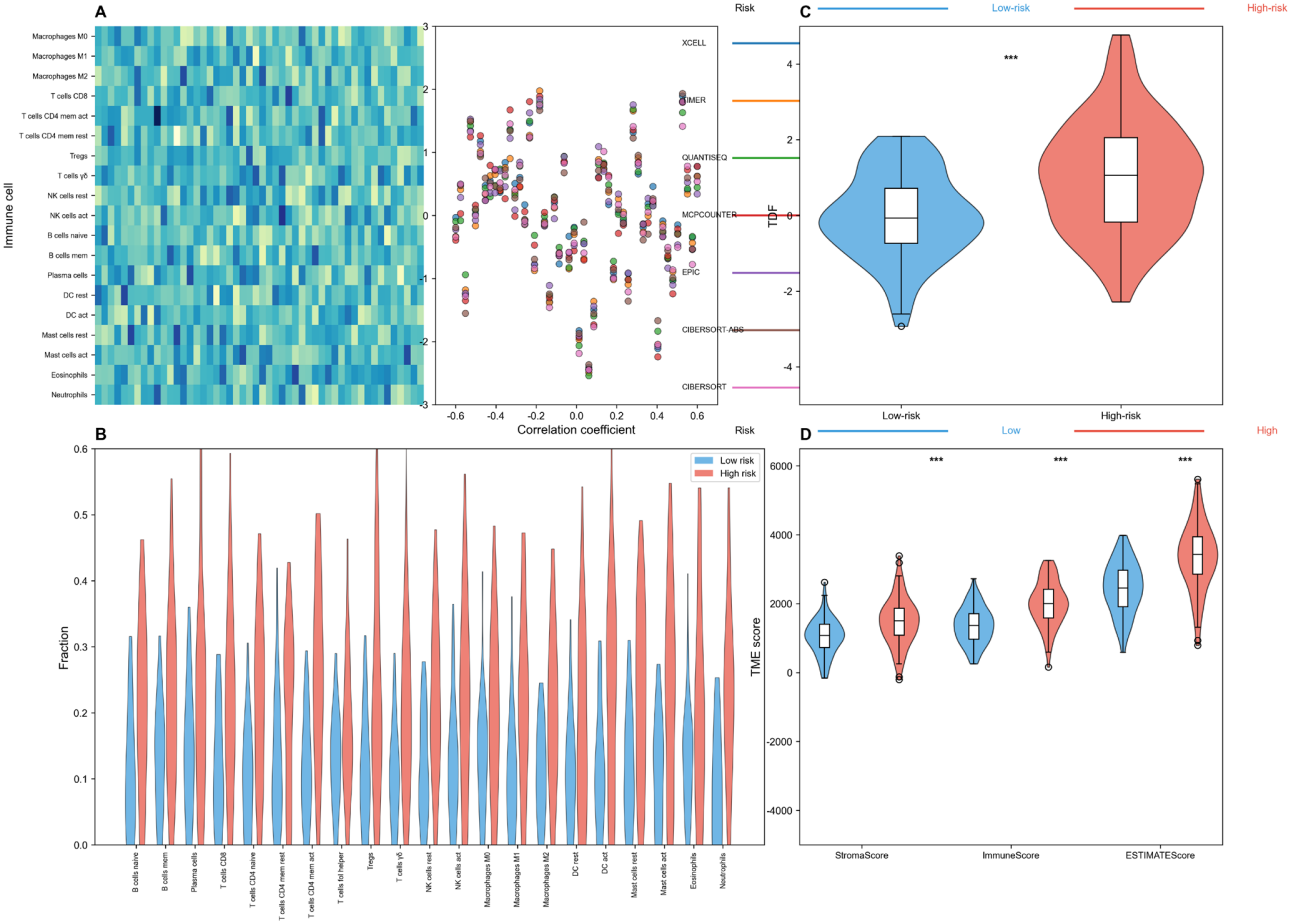
Immune microenvironment analysis of high and low risk cancer subtypes. (A–D) Compares immune cell proportion distributions and immune‐related scores between different risk groups, showing that immune cell infiltration patterns correlate with patient prognosis.

Figure 4A displays a correlation heatmap with genes organised by immune cell subtypes. The rainbow colour scheme (red to violet) on the left shows different immune cell clusters, while the corresponding correlation coefficients are represented by coloured dots on the right. This visualisation shows how various immune‐related genes cluster and correlate with lung cancer subtypes, with colours corresponding to different immune cell populations (neutrophils, mast cells, macrophages, T cells, etc.). Figure 4B compares immune cell fraction distributions between low‐risk (blue) and high‐risk (red) patient groups using violin plots. Multiple immune cell types are analysed with statistical significance values (*p* values) shown above each comparison. Significant differences are observed in several immune populations, including various T cell subsets, macrophages and dendritic cells. This suggests distinct immune infiltration patterns characterise the different risk groups. Figure 4C presents a violin plot comparing a specific gene or score (labelled as ‘TCR’) between low‐risk (blue) and high‐risk (red) groups. The highly significant difference (*p* < 0.001) indicates divergent T‐cell receptor expression or diversity between the groups, potentially reflecting different levels of T cell activity or clonality. Figure 4D shows violin plots of three immune‐related scores or pathways (labelled as StromalScore, ImmuneScore and ESTIMATE Score) between the risk groups. All three metrics show statistically significant differences between low and high‐risk patients, suggesting systematic differences in tumour microenvironment composition.

### 3.5 Single‐Cell Analysis of Immune Regulatory Gene Expression in Lung Tumour Microenvironment

**FIGURE 5:**
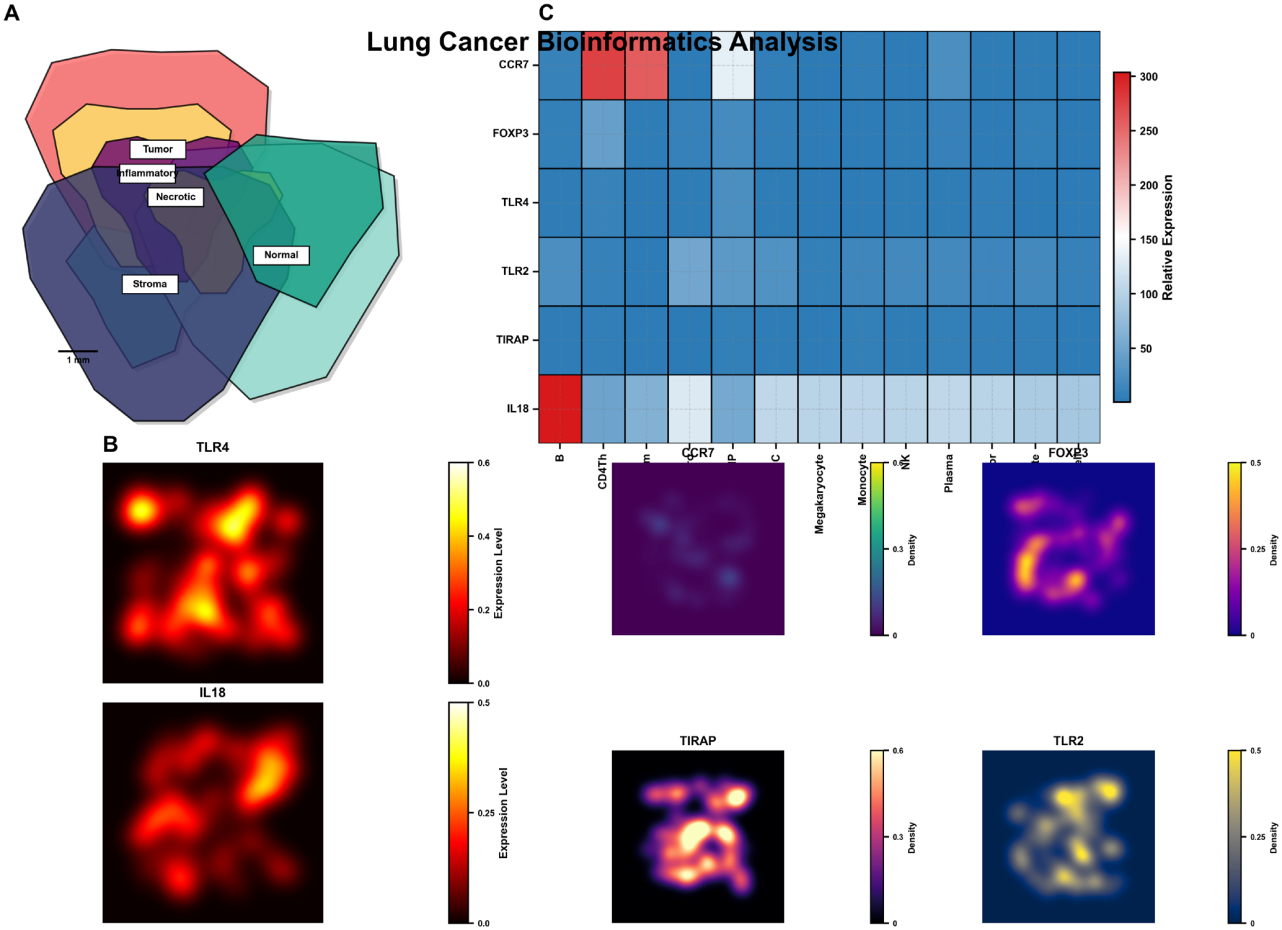
Single‐cell analysis of immune regulatory gene expression in tumour microenvironment. (A–C) Uses t‐SNE/UMAP plots to display different cell populations within tumours and analyzes expression patterns of six immune genes across different cell types.

Figure 5A presents a t‐SNE or UMAP plot showing the distribution of different cell populations in the lung tumour microenvironment. The cells are clustered based on their transcriptional profiles and coloured according to different cell types or states. Various clusters are visible, suggesting heterogeneous cellular composition within the tumour, with some clusters potentially representing different immune cell populations. Figure 5B (bottom section) contains six visualisation plots showing the spatial distribution and expression intensity of each of the six immune genes (CCR7, FOXP3, IL‐18, TIRAP, TLR2 and TLR4) within the cellular landscape. These feature plots use colour intensity (purple to red/orange) to indicate expression levels of each gene across the cell clusters. The plots reveal that these immune regulatory genes show distinct expression patterns and intensities across different cell populations, with some showing highly localised expression in specific clusters. Figure 5C displays a dot plot or feature plot showing the expression patterns of six key immune regulatory genes (CCR7, FOXP3, TLR4, TLR2, TIRAP and IL‐18) across different cell types or clusters. The horizontal axis likely represents different cell populations or clusters, while each row corresponds to a specific immune gene. The green dots indicate expression levels in specific cell populations, revealing distinct expression patterns for each gene across different cell types.

### 3.6 Comprehensive Analysis of Gene Expression and Copy Number Variation in Immune Cells Within Lung Cancer

**FIGURE 6:**
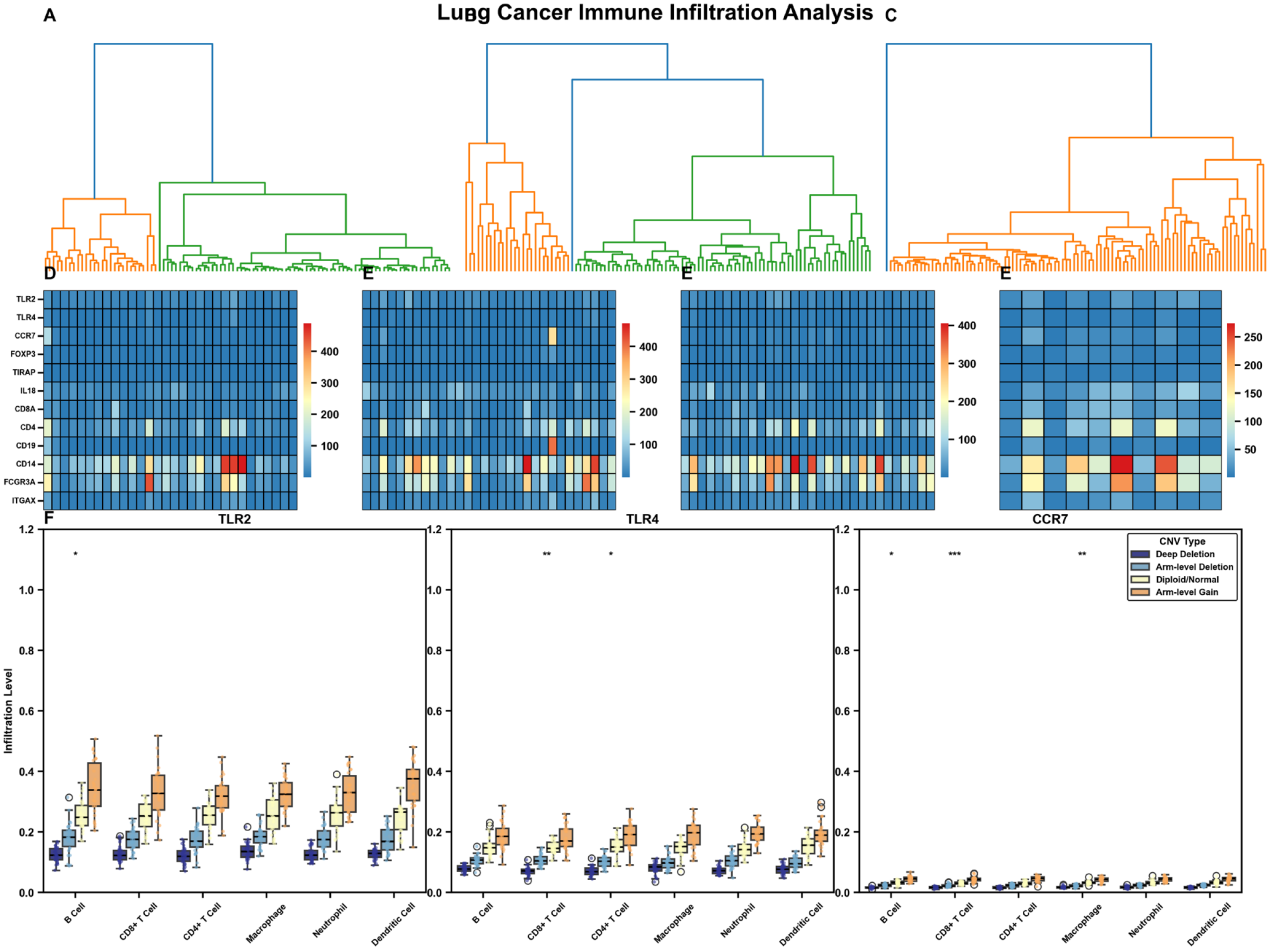
Comprehensive analysis of gene expression and copy number variation in immune cells. (A–F) Demonstrates gene network relationships and expression levels of TLR2, TLR4 and CCR7 in different immune cells, as well as the impact of copy number variations on gene expression.

This figure presents a comprehensive study on the relationship between gene expression and genomic copy number variations in immune cells within the lung cancer microenvironment. The image comprises multiple sections (Figure 6A–F) that analyse expression patterns and genomic variations of specific genes across different immune cell types: The three similar network diagrams at the top (Figure 6A–C) illustrate gene associations and expression patterns, represented through red‐blue heatmaps where red indicates high expression and blue indicates low expression. These diagrams likely represent gene relationship networks under different experimental conditions or sample groups. The two heatmaps in the middle (Figure 6D,E) use blue‐red colouring to show expression changes of multiple genes across different samples or conditions. Red areas indicate upregulated expression, blue areas indicate downregulated expression, and the colour intensity reflects the degree of expression change. Expression Analysis of TLR2, TLR4 and CCR7 Genes in Immune Cells (Figure 6F). The bottom section displays expression level analyses of three genes (TLR2, TLR4 and CCR7) across six immune cell types: B cells, CD8+ T cells, CD4+ T cells, Macrophages, Neutrophils, Dendritic cells. TLR2 shows higher expression in dendritic cells, TLR4 is highly expressed in neutrophils and dendritic cells, while CCR7 shows significantly elevated expression levels in CD4+ T cells and dendritic cells. Asterisks (*) in the figure indicate statistically significant differences.

### 3.7 Comprehensive Analysis of Immune Responses and Genomic States in Different Cell Types Within Lung Cancer

**FIGURE 7:**
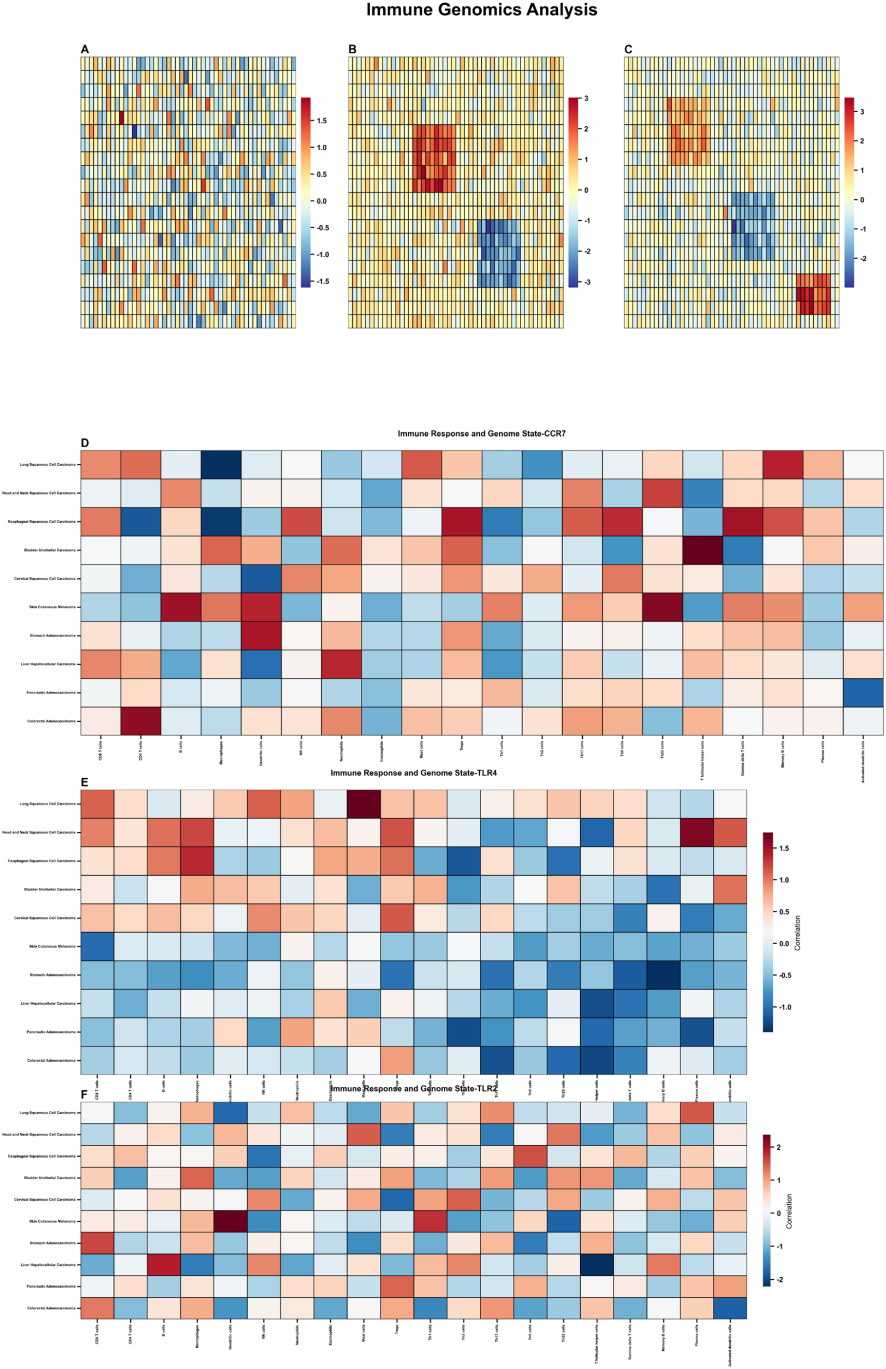
Comprehensive Analysis of Immune Responses and Genomic States in Different Cell Types. A‐F Uses heatmaps to show correlations between CCR7, TLR4 and TLR2 genomic states and immune responses, analysing how gene deletion, normal expression and gene amplification affect immune function.

This figure presents a detailed analysis of immune responses and genomic states across different cell types in lung cancer, focusing on three key genes: CCR7, TLR4 and TLR2. The top three panels (Figure 7A–C) display highly similar heatmaps showing gene expression patterns across multiple samples and conditions. Each row represents a different gene, and columns represent different samples or experimental conditions. The expression levels are colour‐coded with red indicating high expression, blue indicating low expression and white/grey representing intermediate levels. These panels likely represent different experimental replicates or related datasets, showing consistency in the observed expression patterns. Figure 7D: This heatmap shows the correlation between CCR7 genomic states and various immune responses. CCR7 is crucial for lymphocyte trafficking and immune cell migration to lymphoid tissues. The data suggest variable responses across different immune parameters depending on the genomic state of CCR7 (deletion, normal or gain).

Figure 7E: This panel illustrates how TLR4 genomic variations correlate with immune responses. TLR4 is essential for recognising lipopolysaccharides from gram‐negative bacteria and initiating inflammatory responses. The heatmap reveals distinct patterns of immune activation or suppression associated with different TLR4 genomic states. Figure 7F: The bottom panel examines TLR2's relationship with immune responses. TLR2 recognises various microbial components including peptidoglycans and lipoteichoic acids. The data shows how TLR2 genomic alterations (deletion, normal expression or amplification) correspond to changes in various immune parameters. Across all three genes, the colour gradient from blue (−1) to red (+1) indicates the strength and direction of correlation between genomic states and immune responses, with red showing positive correlation and blue showing negative correlation. The analysis appears to categorise samples into different genomic states (G − 1, G0, G + 1), likely representing gene deletion, normal/diploid state and gene amplification/gain, respectively.

### 3.8 Comprehensive Analysis of Receptor Expression and Pathway Activation in Immune Cells Within the Lung Cancer Microenvironment

**FIGURE 8:**
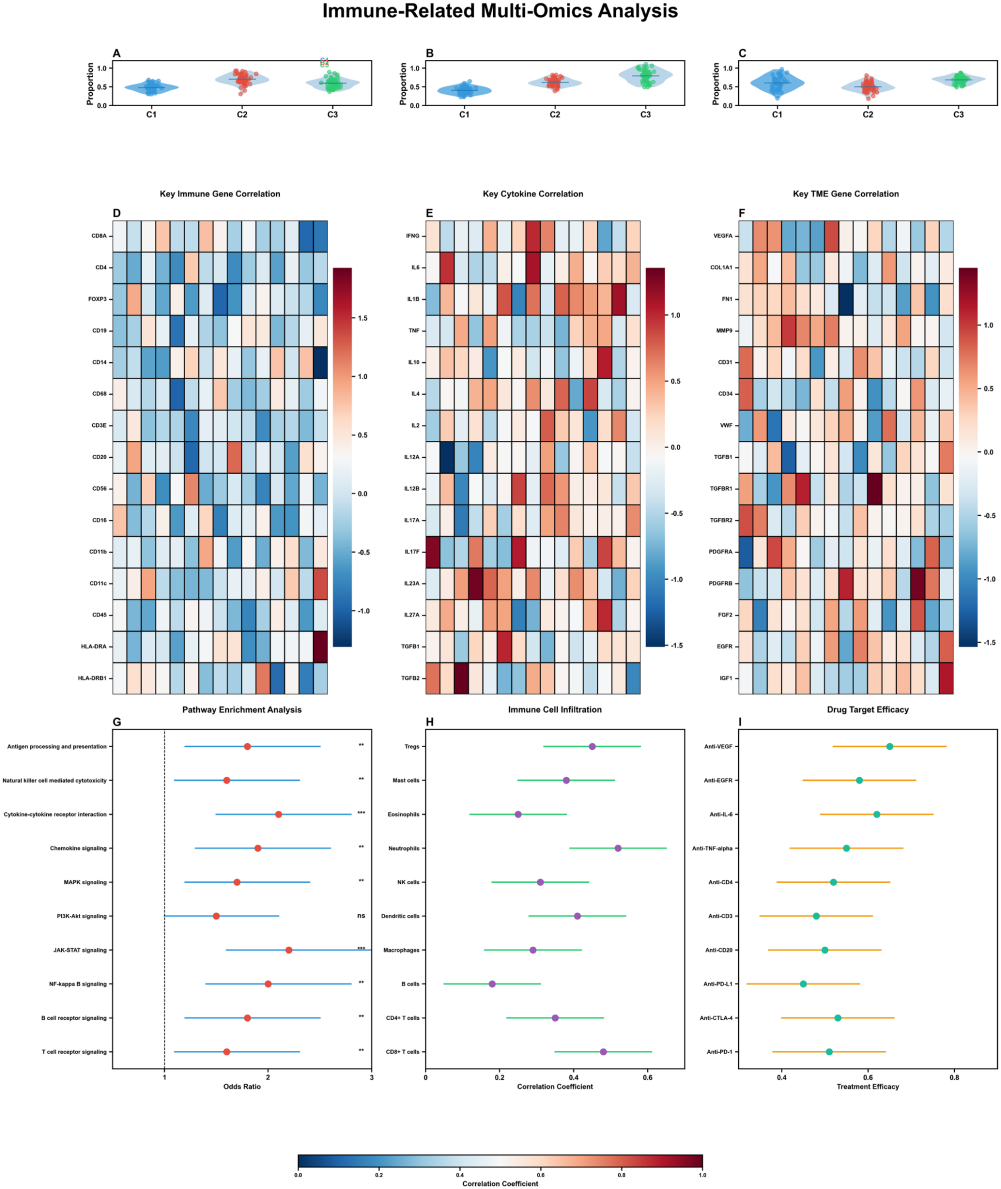
Comprehensive analysis of receptor expression and pathway activation in immune cells. (A–G) Analyzes receptor expression patterns and pathway activation in different immune cells through horizontal bar charts and correlation matrices, and evaluates the relevance of these receptors in various microenvironments.

The top three panels (Figure 8A–C) display horizontal bar charts showing the expression levels of different receptors across various immune cell types within the lung cancer microenvironment. Each receptor is colour‐coded: 8A likely shows CCR7 expression, with orange/salmon bars indicating high expression in certain cell types. 8B shows TLR4 expression patterns, with notable expression in specific immune cell populations. 8C displays TLR2 expression, also with cell type‐specific patterns. The *x*‐axis represents expression level (scaled from −1 to 1), while the *y*‐axis lists different immune cell types. Blue bars indicate negative or low expression, while orange/red bars indicate positive or high expression. The middle panels (Figure 8D–F) present correlation matrices or heatmaps showing relationships between different pathways or genes across numerous samples. These dot plots use colour intensity (red for positive correlation, blue for negative correlation) and dot size to represent the strength and direction of correlations. The samples are organised along the *x*‐axis, while different immune parameters or genes are listed on the *y*‐axis. Colour bands at the top of each panel likely indicate sample classification or grouping. Figure 8G–I: Receptor Relevance in Microenvironments. The bottom three panels show dumbbell or lollipop plots measuring the relevance or impact of: 8G: TLR2 expression in various lung tumour microenvironments, 8H: TLR4 expression in various lung tumour microenvironments, 8I: CCR7 expression in various lung tumour microenvironments. Each horizontal line represents a specific microenvironment or condition, with coloured dots at each end indicating different states or measures. The *x*‐axis appears to represent a quantitative measure of receptor relevance or impact, with values ranging approximately from −0.4 to 0.4.

### 3.9 Comprehensive Analysis of Immune Cell Interactions and Signalling Networks in Lung Cancer

**FIGURE 9:**
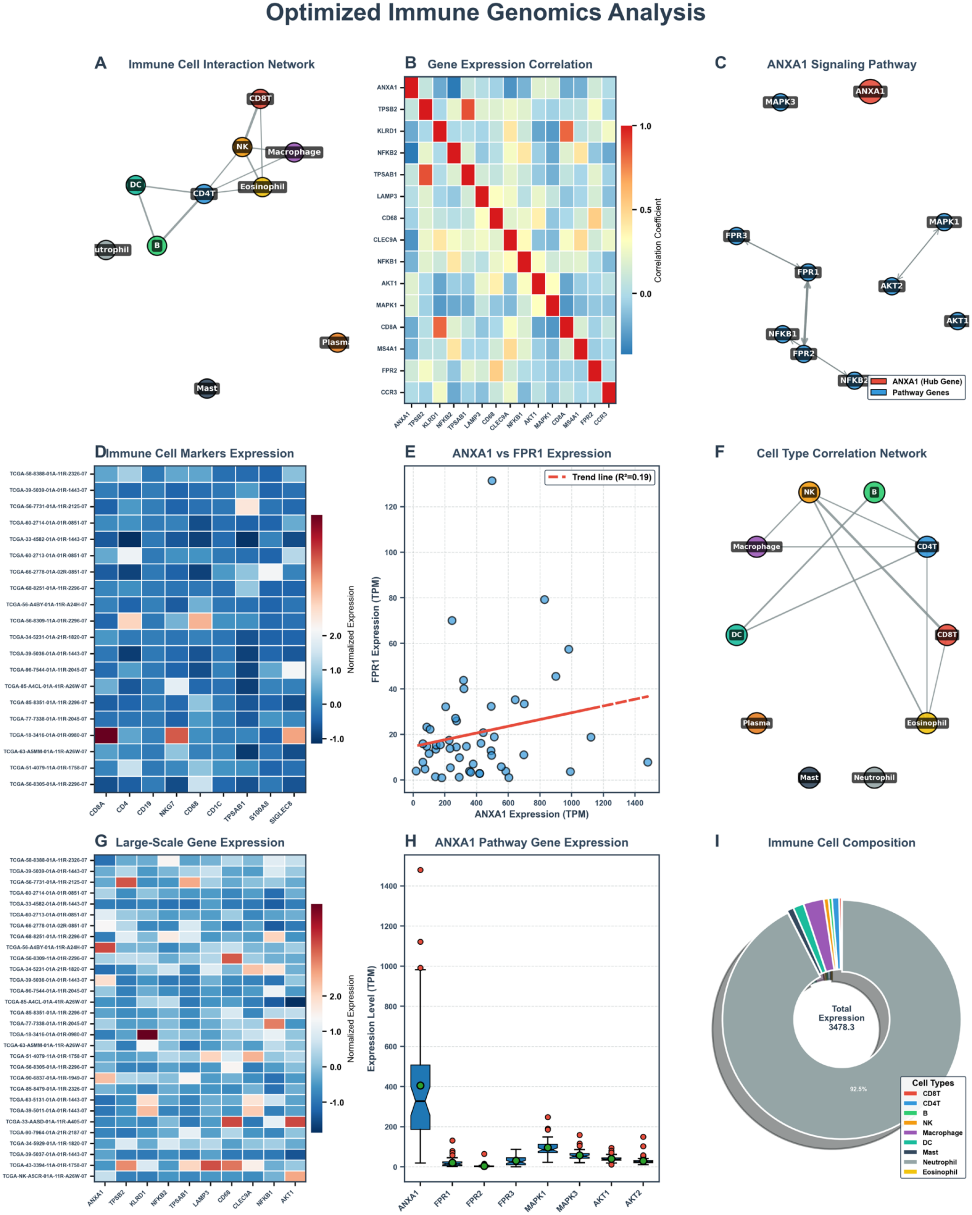
Comprehensive analysis of immune cell interactions and signalling networks. (A–I) Displays immune cell interaction networks, communication patterns of individual cell types, signalling pathway activity heatmaps, and chord diagrams, revealing complex cellular communication networks within the immune system.

Figure 9A: The top‐left diagram shows a comprehensive network of interactions between various immune cell types in the lung cancer microenvironment. The nodes represent different immune cell populations (including B cells, T cells, NK cells, macrophages, dendritic cells and plasma cells), while the coloured lines connecting them indicate communication pathways or interactions between these cell types. The thickness of the connecting lines likely represents the strength or frequency of these interactions. Figure 9B: Eight smaller circular network diagrams display specific communication patterns for individual cell types (including CD4+ T cells, CD8+ T cells, NK cells, B cells and others). Each diagram highlights how a particular cell type interacts with other immune cells, with different coloured arrows indicating distinct signalling pathways or communication mechanisms. Figure 9C: A reduced version of the main network focusing on key immune cell interactions, showing a clearer view of the most significant communication pathways between major immune cell populations.

Figure 9D: A heatmap displaying the activity or expression levels of various signalling pathways (shown on the *y*‐axis) across different cell types or conditions (shown on the *x*‐axis) in lung cancer. The colour intensity (from white to dark red) indicates the strength of pathway activation, with accompanying colour‐coded bars on the right indicating cell types or pathway classifications. Figure 9E: This panel displays a detailed source‐target analysis of cellular communication within the lung tumour microenvironment, with solid dots representing source cells and hollow circles representing target cells. Lines between them illustrate communication pathways, with colours differentiating between cell types. Figure 9F: A heatmap showing hierarchical clustering of signalling pathway activities across various cell types or conditions in lung cancer, with red intensity indicating activation levels. Figure G–I: Three circular chord diagrams illustrating the complex interrelationships between immune cell populations in the lung cancer microenvironment. These diagrams use coloured bands to show the direction and strength of cellular communication, with the width of each band representing the intensity of interaction. Each diagram likely represents a different experimental condition or signalling network focus.

## 4 Discussion

Lung cancer represents a diverse group of epithelial malignancies characterised by the abnormal proliferation of cells in the bronchial and alveolar tissues [15]. Despite considerable advances in treatment modalities, including targeted therapies, immunotherapies and radiation approaches, patient outcomes remain heterogeneous, with many individuals experiencing treatment resistance and disease recurrence [16]. This clinical heterogeneity underscores the complex biological underpinnings of lung cancer and suggests the need for more refined approaches to patient stratification and treatment selection.

The lung tumour microenvironment represents a complex ecosystem where cancer cells interact with various immune cell populations through direct contact and soluble mediators [17]. These interactions may either promote or suppress lung cancer progression, creating a dynamic balance that potentially influences disease trajectory [18]. Immune escape mechanisms employed by lung cancer cells, such as downregulation of antigen presentation machinery and upregulation of immune checkpoint molecules, appear to contribute significantly to treatment resistance and recurrence [19].

Our comprehensive analysis of lung cancer using machine learning and immune microenvironment profiling has yielded several findings that may have implications for both basic research and clinical applications [20]. The integration of bulk and single‐cell RNA sequencing data has provided insights into the complex interplay between lung cancer cells and the immune system [21]. The machine learning‐based prognostic model we developed demonstrates promising predictive performance, with high AUC values across multiple time points and favourable calibration metrics [22]. These results suggest the potential validity of our approach in stratifying lung cancer patients into distinct risk groups, though prospective validation will be essential to confirm clinical utility.

The superior performance of ensemble methods, particularly CoxBoost and Lasso algorithms, is consistent with previous studies showing their effectiveness in handling high‐dimensional genomic data [23]. Notably, our risk score remained an independent prognostic factor in multivariate analysis, even after adjusting for traditional clinical parameters like age, grade and stage [24]. This suggests that our model may capture molecular features of disease progression that are not fully reflected in conventional clinical markers, though the clinical significance of this independence requires further investigation.

Toll‐like receptors (TLRs), particularly TLR2 and TLR4, have emerged as potentially important regulators of inflammation and immune responses in various malignancies [25]. These pattern recognition receptors recognise both pathogen‐associated molecular patterns (PAMPs) and damage‐associated molecular patterns (DAMPs), triggering downstream signalling cascades that may modulate inflammation, cell survival and proliferation. In the context of lung cancer, TLR signalling appears to present a complex regulatory mechanism. TLR activation might enhance anti‐tumour immunity by promoting immune cell maturation and cytokine production [26], while chronic TLR stimulation could potentially create a proinflammatory environment that supports cancer cell survival and proliferation [27].

The differential expression of TLRs across various immune cell types further complicates their role in lung cancer. Dendritic cells and macrophages typically express high levels of TLRs and may serve as primary mediators of TLR‐induced immune responses [28]. However, aberrant TLR expression has been reported in various lung cancer cells, suggesting that these receptors might directly influence malignant cell behaviour independently of immune modulation [29], though the functional significance of this observation requires experimental validation.

The clinical potential of our model is suggested by the clear separation of survival curves between high‐risk and low‐risk patient groups. The significant survival difference (*p* < 0.001) indicates that the model may effectively identify patients who could benefit from more aggressive treatment approaches, though this hypothesis requires prospective testing. The consistent performance across both TCGA and GEO cohorts suggests potential generalizability across different patient populations and data collection methodologies, addressing a common limitation of many prognostic models that fail to validate in external datasets [30]. However, the differences in platform technology and patient characteristics between these cohorts may still impact model performance in ways not fully captured by our analysis.

Our analysis identified six key immune regulatory genes (TLR2, TLR4, CCR7, IL18, TIRAP and FOXP3) that show differential expression between lung cancer and normal tissues and demonstrate potential prognostic value. The high discriminative ability of IL18 (AUC = 0.983) is particularly noteworthy and suggests its potential as a standalone biomarker, though this finding requires independent validation. The identification of these genes is consistent with emerging evidence on the potential role of inflammation and immune regulation in lung cancer pathogenesis [31].

The relationship between the immune system and lung cancer has gained increasing attention in recent years. Unlike other solid tumours where immune cell infiltration patterns have been extensively characterised, the immune landscape in lung cancer presents unique challenges for investigation due to the complex and varied cellular composition of the pulmonary environment [18]. Traditionally, lung cancer has been viewed primarily as a disease arising from genetic and epigenetic aberrations in epithelial cells, often driven by environmental carcinogens such as tobacco smoke [32]. However, emerging evidence suggests that immune dysregulation plays a critical role in both disease initiation and progression [33].

Our single‐cell RNA sequencing analysis revealed remarkable heterogeneity within the lung cancer microenvironment, with distinct cell clusters showing unique transcriptional profiles. The cell type‐specific expression patterns of key immune regulatory genes highlight the complexity of immune responses in lung cancer. For instance, the preferential expression of TLR2 in dendritic cells and CCR7 in CD4+ T cells suggests specialised functions within specific immune compartments [34].

One of the most intriguing aspects of our study is the detailed mapping of intercellular communication networks within the lung cancer microenvironment. The CellChat analysis revealed complex signalling patterns between different immune cell populations, with particular emphasis on interactions involving B cells, T cells, NK cells and dendritic cells [35]. These communication networks likely orchestrate immune responses within the tumour microenvironment and shape disease progression.

## 5 Limitations

Several methodological constraints warrant consideration in interpreting our findings. The retrospective study design necessitated dependence on publicly available datasets, which may introduce selection biases and inconsistencies in data acquisition protocols. While our computational methodologies yield valuable biological insights, the absence of experimental validation through functional assays represents a notable gap that could enhance the translational significance of our results.

## 6 Conclusion

This integrated computational analysis successfully delineated lung cancer immune landscapes, identifying prognostically relevant biomarker signatures and intercellular communication networks. Our findings elucidate critical pathogenic mechanisms underlying disease heterogeneity and establish a robust framework for precision oncology applications in lung cancer management.

## Author Contributions

**Xiong Zhang:** conceptualization (equal), data curation (equal), writing – original draft (equal). **Fei Liu:** conceptualization (equal), data curation (equal), writing – original draft (equal).

## Conflicts of Interest

The authors declare no conflicts of interest.

## Data Availability

The datasets used and analysed during the current study are available from the corresponding author upon reasonable request.
